# From Actions to Effects: Three Constraints on Event Mappings

**DOI:** 10.3389/fpsyg.2018.01391

**Published:** 2018-08-14

**Authors:** Peter Gärdenfors, Jürgen Jost, Massimo Warglien

**Affiliations:** ^1^Department of Philosophy, Lund University, Lund, Sweden; ^2^Innovation and Enterprise Research Lab, University of Technology Sydney, Ultimo, NSW, Australia; ^3^Max-Planck-Institut für Mathematik in den Naturwissenschaften, Leipzig, Germany; ^4^Santa Fe Institute for the Sciences of Complexity, Santa Fe, NM, United States; ^5^Department of Management, Università Ca’ Foscari Venezia, Venice, Italy

**Keywords:** events, monotonicity, continuity, convexity, conceptual spaces, causation, generalization, force

## Abstract

Events can be modeled through a geometric approach, representing event structures in terms of spaces and mappings between spaces. At least two spaces are needed to describe an event, an action space and a result space. In this article, we invoke general mathematical structures in order to develop this geometric perspective. We focus on three cognitive processes that are crucially involved in events: causal thinking, control of action and learning by generalization. These cognitive processes are supported by three corresponding mathematical properties: monotonicity (that we relate to qualitative causal thinking and allows extrapolation); continuity (that plays a key role in our activities of action control); and convexity (that facilitates generalization and the categorization of events, and enables interpolation). We define how such properties constrain events representations and relate them to thinking about events. We discuss the relevance of the three constraints for event segmentation and explore the implications of such constraints for semantics. We conclude by a discussion that relates our approach to other accounts of events.

## Introduction

Events lead a double life, Davidson (1967) remarked more than 40 years ago. They still do. We refer with ease to actions and events in ordinary talk, and reason without effort about them. Still, their conceptual structure and the way we use it for our basic cognitive activities remain to a large amount an open issue (Pianesi and Varzi, 2000; Zacks and Tversky, 2001).

While notions of events in philosophy and science may diverge, they all agree that events involve participants and relations between them (Casati and Varzi, 2008). Thus events are relational concepts (Gentner and Kurtz, 2005). Yet the nature of such relations has not been systematically analyzed.

In earlier work (Gärdenfors and Warglien, 2012; Warglien et al., 2012a; Gärdenfors, 2014), we have suggested a geometric approach to event conceptualization and shown how the semantics of verbs provide a fundamental window over the geometric structure of event concepts. The key idea is to represent event structures in terms of spaces and mappings between spaces. At least two spaces are needed to describe an event, an action space and a result space. For us, the temporal structure of events is not defining, but it emerges from the interactions of the components of an event. This is in contrast, for example, with Zacks and Tversky (2001) that focus on the temporal structure of events. For them an archetypical event is “a segment of time at a given location that is conceived by an observer to have a beginning and an end” (Zacks and Tversky, 2001, p. 3).

In this article, we invoke general mathematical structures (Jost, 2015) in order to develop this geometric perspective by analyzing three important properties of event mappings that constrain the structure of the event and support fundamental cognitive uses of event concepts. We focus on three cognitive processes that are crucially involved in thinking about events: causal thinking, control of action and learning by generalization. These cognitive processes are supported by three corresponding mathematical properties: monotonicity (that we relate to qualitative causal thinking); continuity (that plays a key role in our activities of action control); and convexity (that facilitates generalization and the categorization of events). We argue that these constraints play a central role in the ‘working model’ of an event (Zacks et al., 2007; Radvansky and Zacks, 2014). In mathematical terms, monotonicity rests on an order structure, continuity is a topological notion depending on a notion of proximity, and convexity is a geometric concept, utilizing the notion of betweenness.

In Section “A Two-Vector Representation of Events,” we briefly summarize the building blocks of a geometric model of events, the space representing the forces generating the events, and the result space representing changes induced by such forces. An event is specified by the mapping between these spaces. In Section “Three Qualitative Principles for Events,” we describe the three properties that constrain such a mapping. These constraints are supported by research on naive physics, qualitative causal reasoning, action perception, psychology of motor control, and cognitive linguistics. In Section “Mathematical Correspondences of the Constraints,” we define the mathematical correspondences to the constraints. Sections “Event Structure and Semantics” and “Manner and Result Verbs” explore the implications of such constraints for semantics. Our paper does not directly focus on the problem of event segmentation that has been in focus in much of research on events (e.g., Radvansky and Zacks, 2014). However, we discuss the relevance of the three constraints for this area in Section “Contributions to Working Models and Segmentation.” We conclude by a discussion that relates our approach to some other accounts of events.

## A Two-Vector Representation of Events

When we talk about events, we clearly imply a relational structure. For example, linguists resort to a thematic role structure where the basic roles are agent and patient (Dowty, 1991; Levin and Hovav, 2005). When we think about everyday physical phenomena, we conceive of events as relations between causes and effects. When we make plans, we consider different actions and their consequences. These three perspectives reveal different aspects of the same underlying structure, which is an asymmetric relation between two entities.

This abstract common structure of events in language, physical thinking and planning can be naturally interpreted in geometric terms. The structure can be modeled as a mapping between vector spaces. The action and the result components of the event can then be represented as vectors in the two spaces.

Such a geometric perspective on events can be found in the work of several authors (e.g., Talmy, 1988; Wolff, 2007, 2008, 2012; Croft, 2012; Gärdenfors and Warglien, 2012). In order to model events, the spaces involved must be characterized more precisely. Building on previous work on conceptual spaces (Gärdenfors, 2000, 2014; Gärdenfors and Warglien, 2012; Warglien et al., 2012a), we characterize both spaces as vector spaces. The action space can be conceived as a space of forces (or force patterns) acting upon some target entity, the properties of which are described in the result space. As the result component of the event represents changes in the properties of the target, the result space can also be modeled as a vector space. The changes are typically changes of location or changes of object properties. For example, when Lucy opens the door, the agent Lucy exerts a force vector (action) on the door that leads to a change of the position of the door (result). Or in the event of the wind bending the antenna, the force of the wind (action) leads to a change of the shape property of the antenna (result). While both spaces have the same geometric structure, they represent different types of vectors: forces have a different nature than changes in properties. In the limiting case when nothing changes, that is, when the result vector is the identity vector, the event is a state.

The target entity of the event is called the *patient* and the result vector describes the changes of the properties of the patient. If there is an entity that generates the action vector it is called the *agent*. There exist, however, events without agents, for example events of falling, drowning, dying, growing and raining.

The mapping between the force space and the result space is what characterizes the event. For example, pushing a cupboard sometimes results in the cupboard moving, sometimes not; shooting a moose sometimes kills it, sometimes not. In such cases the mapping between the force vector and the result vector qualifies two different events.

The nature of event mappings has been little investigated and in our earlier work (Gärdenfors and Warglien, 2012; Warglien et al., 2012a; Gärdenfors, 2014) we have not developed the properties of the mappings. The new contribution of this paper consists of an analysis of three general principles for such mappings that highlight central aspects of human reasoning about events. All three principles can be expressed in qualitative terms, reflecting the qualitative nature of everyday thinking about events.

The event model captures a basic sense of *causation*: the action *causes* the result. Most accounts of causation analyze the relation between the action and the effect as a relation between two events (see e.g., Zacks and Tversky, 2001). In contrast, our model views causation as a relation *within* an event by introducing a distinction between forces and changes of states (cf. Wolff, 2007, 2008, 2012). Unlike many other theories, our model does not treat causes and effects as symmetrical entities: they belong to different domains – causes to the force domain and results to change in location (in the case of movements) or in some property domains (color, size, weight, temperature, etc.).

A geometric approach to events also allows a natural representation of event categories. An event category is often characterized by similarities of results or similarities of actions. For example, events of opening a door cover a large range of different changes in the position of the door. Similarly, pushing a door involves a large range of forces exerted on the door (some of which result in the door moving, some not). These event categories are based on similarities in action or result space. This is in line with a general claim that similarity is fundamental for concept formation (Nosofsky, 1988; Goldstone, 1994; Gärdenfors, 2000). The importance of result and action categories is reflected in the fact that a lexicon typically represents such categories rather than specific events (Warglien et al., 2012a). As we will discuss later, when categories of events involve both similar actions and similar results, for example in jump or dive, similarity also matters when analyzing the mappings between spaces.

While the two-spaces model of events is meant to capture mostly qualitative features of event representations, it can provide more precise and testable propositions (and approximate modeling strategies). For example, experimental studies suggest that individuals can perform intuitive addition of force vectors when observing how simultaneous sources of force affect a patient trajectory (Wolff, 2007). Classical experiments based on Michotte’s “launching” paradigm (Michotte, 1963) show that the attribution of causality in an event depends on the angle of the trajectory of a hit object B with a hitting object A – which demonstrates that individuals decide whether an animation captures a single (causal) event or two disjoint ones on the ground of the mapping of forces to trajectories. Recent research has shown the perception of such mapping to be remarkably accurate, and a good predictor of “causal impression” (White, 2012). In all these cases, simple two-spaces models of events provide good approximate predictions of individual perception of causal events and the satisfaction/violation of such predictions affects the evaluation of what constitutes an event. This paper further specifies relevant constraints on elementary event representation.

## Three Qualitative Principles for Events

Our general theoretical hypothesis is this: Events as such can be arbitrarily complicated. To get a mental grip on them, we break them up into more elementary constituents, and these constituents are assumed to satisfy some general structural constraints. To express those basic constituents, we have appropriate verbs at our disposal. Those verbs model a mapping from a force space into a result space. These spaces can often be assumed to be vector spaces or to have at least properties similar to vector spaces. This metaphor also applies to social interactions where we are confronted with intentions additionally to physical forces. To understand events and apply verbs in situations that interpolate or extrapolate from known ones and to predict the outcome of small variations in the input, we utilize structural constraints. Let us now be more specific.

The core of our model of events is the mapping between the two spaces – the force space and the result space. Although other theories of events (Talmy, 1988; Wolff, 2007, 2008; Croft, 2012) also recognize the importance of such a mapping, they do not analyze it. Here our main aim is to show that, although the mapping is in general not fully specified, it respects some general constraints that still make the model cognitively productive. In this section, we present three constraints that correspond to our understanding of causation, control of action and generalization. In the following section, we show that these constraints have fundamental mathematical expression in terms of monotonicity, continuity and convexity of the mapping.

### Larger Forces Lead to Larger Results (Monotonicity Constraint)

When the outcome of an action is very hard to predict given ignorance of counter-forces and approximate understanding of causal laws, the effect of actions can still be understood in qualitative terms. A very general constraint is that, whatever counter-forces and other forces are present in a given situation, increasing the action force will also increase (or at least not decrease) the magnitude of the effect. For example the effects of pushing an object may vary widely depending on the friction of the surface. Nevertheless, pushing the object harder means that it moves further (or at least does not move in the other direction). This ordinal principle implies a sort of ceteris paribus thinking: Other counter-forces can be of varying magnitude and direction, but no matter what they are, ceteris paribus, the change of effect is predictable from the change of the action force.

Given the unknown nature of the counter-forces, the direction of effect need not be the same as the direction of the force vector. For example an unknown side wind may effect the direction of the movement of a motorboat, so that the direction of the movement driving force of the motor may become oblique in relation to the driving force of the motor direction of the movement (Wolff, 2007). Still the general monotonicity constraint is valid: If the force of the boat motor is increased, the speed of the boat will increase.

The reason the constraint is cognitively important is that it captures our understanding of how a change of an action will change the outcome. This is a basic step in understanding causality (Hume, 1748/2000; Wolff, 2007, 2008) and in making causal inferences. A related motivation is that the monotonicity principle implies at least a qualitative predictability of the effects of action.

Monotonicity can also support the reverse inference process. It has been observed that “an effect is attributed to the one of its possible causes with which, over time, it covaries” (Kelley, 1973). When needing to identify causal factors among multiple potential ones, monotonicity can provide a powerful selection criterion, that captures an ordinal covariation principle. For example, the tides have been observed as long as humans have existed, but it was only when the monotonic correlations to the moon’s position and distances was discovered that we understood the force vectors causing the tides.

Motion events are fundamental for our understanding of the physical world. Yet there are systematic discrepancies between our intuition of motion and its physical reality (McCloskey et al., 1980; Caramazza et al., 1981). These discrepancies reveal how we conceptualize events. In particular, naïve intuitions of motion tend to follow the scholastic notion of impetus developed by medieval natural philosophers in the Aristotelian tradition. The impetus is thought of as keeping the object moving after it has separated from the source of the force. According to the conception, objects slow down and finally stop because the impetus gradually diminishes (Dijksterhuis, 1950; Duhem, 1958; Maier, 1968). By ignoring the counter-forces of resistance, this view preserves a simple monotonic relation between impetus and speed: a diminishing speed is explained by the diminishing impetus. Even when individuals consider counter-forces such as friction, these are in turn interpreted as having a monotonic effect: more effort generates greater results, but greater resistance reduces results. diSessa (1983) has labeled such monotonicity properties “Ohm’s phenomenological primitive” claiming that this primitive is a basic building block of our intuitions about physical reality.

While monotonicity is easily defined in one dimension, it is a more problematic notion when multiple dimensions are involved. Changes in the force vector may involve different directions of change in different dimensions and then monotonicity may not hold. Either process is monotonic, but since they take place along different dimensions, they are not naturally combined into a single monotonic process. Consequently, the monotonicity constraint can be expected to apply only when the changes go in the same direction. In the case of causal inference, this common direction is called “qualitative synergy” (Wellman, 1990).

Monotonicity also enables qualitative causal inferences. First of all, it allows making purely ordinal inferences about the relationship between causes and effects. For example, while different individuals may react with different intensity to a medical treatment, making quantitative predictions difficult, one can still predict that larger doses of the treatment will have larger effects, independently of the person that is treated. Mill (1843) called this form of causal inference “the method of concomitant variations.”

Moreover, if causal relationships are monotonic, inferences over whole chains of cause-effect relationships can be made, allowing assessments of indirect causation. Wellman (1990) suggests two operators that allow to chain causal relations. One is reduction: two chained positive monotonic relations can be reduced to a single one. The other is inversion: a chain of a positive and a negative monotonic relation reduces to a negative one. These operations are easily illustrated. When you press the accelerator pedal of your car harder, the engine operates at a higher speed, and as a result, the car runs faster. These two actions, that of the driver and that of the engine, get reduced to the single one of accelerating the car. In contrast, in order to see inversion, when you want to brake, you step on the brake pedal, and the brakes then decelerate the car. Thus, there is a positive action, activating the brake, and a negative one, reducing the speed of the car, and the two actions are reduced to the single negative one of braking the car. As both operators reduce a chain of two relations to a single one, they can be used iteratively to reduce even longer chains of relations.

The chaining of causes plays an important role in the structure of events that involve instruments. Actions involve in many cases instruments, and it is not uncommon to represent the full event through a compressed version of it. For example, we can define an event as “the hammer broke the window” although we understand that some agent broke the window through the instrumental use of a hammer. We submit that such typical compression of instrumental events is made possible by the chaining of causal effects which is enabled by monotonicity. By transferring its force to the instruments, the agent endows the instrument of a kind of derived agency, which allows the construal of a reduced representation of the event itself.

Monotonicity applies also to non-physical types of events, of a more social or moral nature. For example, more unfair offers will cause more resentment. Again, monotonicity makes it easy to compose a chain of causal structures: more greed will cause more unfair offers that will cause more resentment, which can be contracted to “more greed will cause more resentment.”

Obviously, not every relation between cause and effect is monotonic. We only claim that the relation between the exerted *force* and its effect is typically monotonic. To understand this better, let us discuss the example of rotations. Here, the motion is obviously non-monotonic. The only monotonic relation is that between the exerted force and the angular velocity. In fact, the mental effort involved in the processing of rotations is an increasing (approximately linear) function of the rotation angle, see e.g., Ellis and Young (1988) and the discussion in Breidbach and Jost (2006). No such phenomenon seems to occur for translation movements. This indicates that the non-monotonic nature of rotations as opposed to that of translations requires an increased mental effort – which, by the way, is monotonically increasing itself.

When throwing an object, the distance achieved is a non-monotonic function of the horizontal angle. But this does not contradict our general principle that the effect is a monotonic function of the force applied. For the same angle, the distance increases as a function of the force with which the object is thrown.

Also, many optimization problems appear to be non-monotonic in nature. In economic optimization, the profit achieved is the product of the price per unit and the number of units sold. The latter, however, typically is a decreasing function of the price per unit. Thus, non-monotonicity here results because a monotonically increasing function is multiplied by a decreasing one. That is, in order to see the monotonicity, we need to break up the effect into more elementary constituents.

### Small Changes in the Force Lead to Small Changes of the Result (Continuity Constraint)

The continuity constraint says that small changes in force should lead to small changes of the result. The constraint is an expression of our sense of control. When we want to change the effect only by a small degree, we can achieve that by applying a sufficiently small change of effort. When we turn the steering wheel a little more to the right, we expect the car also to move a little more to the right, and not to make a U-turn. When we hit the tennis ball a little harder, it will fly a little faster and further, but not move wide out of the court. And when we boil the egg a little longer, it will become somewhat harder. Large changes in force could still lead to small changes of the result, but, expressing it in qualitative terms, the continuity constraint forbids that small force changes produce large effect changes.

The continuity constraint is more demanding in terms of structure, since it requires more than ordering relations (see next section). This structural cost comes with a benefit since it allows finer predictions of the causal effects of actions. In particular, this makes the effects of our actions quantitatively controlled. This is clearly important for learning many motor skills where by fine-tuning our actions we want to modify the effect to a precise degree. Monotonicity alone would not be sufficient here, because that would tell us only that a stronger effort would lead to a stronger result, but not help us in predicting to what extent we need to increase our effort in order to achieve a precise degree of increase of the effect.

Motor control, in general, requires the fine-tuning of an agent’s forces (Wolpert and Flanagan, 2001). For example, balancing a pole requires very small adjustments in the neighborhood of the equilibrium position. Another example concerns how people unintentionally coordinate their actions as when they walk at the same pace. A fascinating laboratory example concerns two persons sitting in two rocking chairs with different natural rocking frequencies (Richardson et al., 2007). The experiment shows that individuals spontaneously coordinate their rocking frequencies in patterns closely resembling classical coupled oscillator dynamics. This illustrates well the importance of continuous adjustments in motion events. If the adjustments were not continuous, it would be difficult to achieve coordinated movements.

Intuitively, continuity corresponds to drawing or tracing a line without jumps. The precise mathematical formulation involves the concept of a limit, and it is usually expressed in terms of Weierstrass’ ε-δ formalism. In fact, continuity can be expressed in purely topological terms. We only need a qualitative notion of proximity (see Continuity). Intuitively speaking, a mapping is continuous if whenever one wants to have similar effects, then it suffices to make forces sufficiently similar.

While the continuity constraint is a very general organizing principle, it is important to realize that it does not always apply. In fact, the cases where it does not apply need a treatment of their own. Consider the example where you are stretching a rubber band. The initial results of increases in force will be continuous changes in length, but eventually a critical point will be reached where the band breaks. In more general terms, we see here a discontinuous phase transition occurring when an obstructing counterforce is suddenly overcome, and a qualitatively new result is achieved. Thus, at or near the transition point, a very small change of effort produces a large result, and the mapping from force to effect is discontinuous. The monotonicity constraint, at least when not interpreted in the strict sense, i.e., that strictly larger forces lead to strictly larger results, will still apply. Monotonicity is a qualitative principle that neither needs continuity nor a quantitative substrate as provided by a metric.

In some cases, such a discontinuous phase transition may mark a cognitive separation between two different events, like handling or destroying something, cooking or burning, bending or breaking. There is an asymmetry here, however. We can bend a twig to a varying degree, but when we break it, this results in a single state that is no longer amenable to a degree of quantification. Therefore, a more fruitful cognitive perspective seems to consist in distinguishing between continuous and discontinuous events.

As we shall elaborate in Section “Applications,” verbs expressing continuous events can be modified by adverbs that serve to express the degree of the effort or force applied. No such modification, however, is possible for verbs that express discontinuous phase transitions. Such verbs typically express the transition between only two possible states, that before the force is applied and that resulting from its application. Before you smash the window, it is intact, and after you have smashed it, it is broken. Neither state is considered to be amenable to a degree of quantification. This is a binary situation. In contrast, for continuous events, a continuum of possible degrees and states is assumed.

Again, the constraint of continuity extends well beyond the domain of physical events. In some types of phenomena, like most economic ones, assumptions of continuity are very common. For example, most market events commonly satisfy the constraint of continuity. However, there may be cases in which straightforward analysis can be challenging due to the heterogeneity of the factors involved. For example, historical events present a mixture of continuity and discontinuity along all dimensions of social structure. Events such as revolutions (Sewell, 1996) are highly multidimensional events where cascading transformations of many dimensions, located in different moments, coexist with the continuity of other domains. Indeed, the choice of the scope of discontinuities is often used to draw the boundaries of the event itself (see again Sewell, 1996). It is striking that most of the time such complex events are expressed through their projection on simple (and often metaphoric) representations in which the continuity constraint – or its violation – carries important structural information about the most salient dimensions. These metaphoric representations usually project these complex multidimensional events on simple physical domains where prototypical notions of continuity and discontinuity are better understood (e.g., the fall off the Roman empire). The use and abuse of such metaphorical projections provides, in our view, strong support to the cognitive cogency of the continuity constraint and its physical prototypical sources.

### Intermediate Results Are Caused by Intermediate Forces (Convexity Preserving Constraint)

An even stronger demand on the cognitive structure of events is to impose a distance measure, i.e., a metric on the spaces involved in the mapping. This allows us to define betweenness and convexity (see Convexity) on the spaces. The convexity preserving constraint on the event mapping *f* can then be formulated as: if the force vector *z* is between force vectors *x* and *y*, in the sense that they all point in the same direction, but the strength of *z* is between those of *x* and *y*, then the result *f(z)* is between the results *f(x)* and *f(y).* In other words, *intermediate forces lead to intermediate results*^1^.

Thus convexity is fundamentally involved in the process of *learning* new concepts (see Gärdenfors, 2000, Ch. 3; Gärdenfors, 2001). From a cognitive point of view, convexity is closely related to *generalization*: It allows us to make inferences over whole regions from a limited number of observations. In general, feedback control mechanisms presume that the mapping from actions to result is convexity preserving. For example, when you are shooting with a cannon you can control the horizontal and vertical direction of the cannon. If you have tried to settings *x* and *y* for the cannon and observed the landing points *f(x)* and *f(y)* of the projectile, you presume that an intermediary setting will lead to an intermediary result. Similar principles apply to many cases of motor control. For example, Runesson and Frykholm (1981) showed subjects movies of a person lifting objects of different weights. The subjects could not see the object themselves but only the movement patterns of the person doing the lifting. Nevertheless, the subjects were very accurate in predicting the weights of the object. This would not be possible, unless the mapping from forces to results (object being lifted) is convexity preserving: From the movement patterns, the subject could infer the forces exerted by the person lifting the box and then (implicitly) infer that intermediary forces corresponded to intermediate weights of the boxes.

In the case of events, the convexity preserving constraint allows to generalize over sets of events and thus to form event categories. The convexity preserving constraint therefore has a fundamental cognitive property: It allows moving from categories of action and categories of result to *categories of events*. More precisely an event category is a convexity-preserving mapping from categories of action to categories of result. For example, in a “jump” event, both action and result are implied. On the force side, a pattern of forces with a vertical component (and gravity as a counterforce). On the result side, a movement with a vertical component. Convexity in this case implies that given two jumps, a jump which is between those in terms of force exerted and direction of the movement will be also called a jump. Of course, events may in general imply too complex mappings between force and result to be captured by a single word, in which case they can be expressed by composing a force and a result expression, like in “driving into town.”

Convexity turns out to be of great importance whenever a problem of categorization or classification of events is at stake also in complex action domains such as those object of the legal domain. The domain of law provides important examples of application of convexity principles to the interpretation of events that do not have just a physical nature. For example, in the contract law, it is often important to determine which type of event happened, in order to know whether a contract has been fulfilled or not. In all these cases, defining a convex range of performances (acts and acts outcomes attributes) within which a given performance happens determines the attribution of the event to one type or the other (for a classical law school case, see Frigaliment vs. BNS).

The three qualitative constraints that we have presented here are organizing principles that govern the mapping from causes to results. They allow general and robust inferences independently of the counter-forces that are present and of the initial conditions of the action. Each qualitative constraint leads to a different type of inference, extrapolation in the case of monotonicity, interpolation for convexity, and effect control for continuity. We view them as principles that considerably improve the cognitive economy of our causal thinking. Like all cognitive simplification strategies, they have their limitations, and there do exist cases where they don’t apply. We expect, however, that such cases require a higher mental effort than those where the principles apply. For instance, boomeranging is a very surprising phenomenon.

The three constraints involve different mathematical structures, as will be described in detail in the next section. Therefore, neither of them can be derived from the others. Here are also some simple examples that they are independent of each other when constraining events. Periodic motions are recurrent and therefore not monotonic (although their speed will typically depend monotonically on the force exerted), but they are usually continuous. The example of a boomerang just mentioned is a more exotic case. – Result verbs usually describe non-continuous effects, called phase transitions in physics. At the critical point, exerting just a little more force may break a window. Monotonicity holds, since utilizing a stronger force on another window will also break it. Convexity also applies in the sense that when two force magnitudes each suffice to destroy a window, any intermediate one will do as well. If one stretches a spring, the elastic energy acquired will depend on the force exerted within a certain range, and in that range, convexity holds. When the force exceeds a critical threshold, however, instead of an elastic, we get a plastic deformation, and monotonicity ceases to hold. More abstractly, while we may still interpolate within a tested range of validity of a phenomenon, where convexity holds, we may not be able to extrapolate beyond that range, and a prediction based on a monotonicity assumption outside that range may not be valid. Whether or not a discontinuous phase transition occurs in such situations, that is, whether continuity holds or fails, is another matter.

## Mathematical Correspondences of the Constraints

Our general thesis for cognitive processes dealing with events is that they are fundamentally guided by some formal principles that are based on general mathematical structures. The structures that we identify here are monotonicity (order), continuity (topology), and convexity (geometry). We shall now describe those structures in turn and explain how they lead to principles that constrain the mapping between forces and results in the event model. A detailed reference for the mathematical concepts is Jost (2015).

### Monotonicity

The first concept is that of an *ordering structure*. For example, time and numbers are ordered, but space is not. An ordering gives us essentially a one-dimensional arrangement. For our purposes, actually, a more general concept suffices, that of a preorder. A preorder is a binary relationship **≤** on a set *X* with the properties that for every element *x*, we have *x* ***≤*** *x*, and if two elements satisfy both *x* ***≤*** *y* and *y* ***≤ x*,** then ***x = y*,** and if we have for three elements ***x ≤ y*** and ***y ≤ z*,** then also ***x ≤ z.*** When we want to have an order, and not only a preorder, we require that for any two distinct elements precisely one of the two relations *x* ***≤*** *y* and *y* ***≤ x*** is satisfied. Thus, with a preorder, we may not be able to decide which of two elements is the larger one, but when we have an order, this can always be decided. In the sequel, we shall assume for simplicity that we have orders, and not only preorders, but the subsequent reasoning applies mutatis mutandis also to preorders.

*Definition 1*: Let *X* and *Y* be ordered sets, with both orderings denoted by the same symbol **≤** . A mapping *f: X→Y* is called *monotonic* if *x* ***≤*** *y* implies *f (x)* ***≤*** *f (y)* for all *x, y* in *X.*

Thus, a monotonic map from *X* to *Y* preserves the ordering. Our first constraint that larger forces lead to larger results corresponds mathematically to that the mapping from forces to results is monotonic.

As explained, an ordering is essentially a one-dimensional structure. In contrast, the space of forces will often have more dimensions. The constraint nevertheless applies, because a force vector will select a direction, and along this dimension, forces can be ordered according to their strength. And if the counterforce is constant, the application of such a directed force should also select a direction in the result space. Thereby, the relation between forces applied in a specific direction and the resulting effects becomes a relation between ordered structures, and the constraint applies: Stronger forces applied in the selected direction lead to stronger results in the enforced direction.

### Continuity

The mathematical structure of a topology turns a set into a space. A set simply is the collection of its elements, and as such it is structureless and amorphous. A topology on a set introduces a notion of nearness or vicinity. A topology on a set distinguishes certain of its subsets as “open.” This is meant to indicate that an open set is a neighborhood for all its members, in the sense that for each element, it also contains the elements surrounding it.

When we have such a notion of vicinity, we can also formulate the concept of continuity as preserving the neighborhood relation:

*Definition 2*: A mapping *f: X→Y* between topological spaces is called *continuous* if the pre-image under *f* of any open subset of *Y* [denoted *f^-1^(Y)*] is an open subset of *X*.

Then we apply continuity to our transformation from force to result: The second constraint that small changes in the force vector lead to small changes of the result vector corresponds mathematically to that the mapping from forces to results is continuous.

The notion of continuity in definition 2 is purely qualitative, basically saying that points that are mapped onto nearby points in *Y* are also nearby in *X*. In order to make it quantitative, one needs a metric, that is, a function that assigns to any two elements of a set their distance. This brings us into the realm of geometry.

A metric induces a topology. With a metric, one can then express nearness (proximity, vicinity) in a quantitative manner. When the topology of a set is induced in that manner by a metric, one can follow Weierstrass and formulate continuity in a quantitative manner. A map *f: X→Y* between metric spaces, with metrics *dX* and *dY*, is continuous at *x* if for every ε > *0* there exists some δ *> 0* such that whenever *dX(x, y) <* δ for any *y* in *X*, we have *dY[f(x), (y)] <* ε. We can then also impose a continuity requirement that simultaneously holds for all points *x* in *X*.

*Definition* 3: A map *f: X→Y* between metric spaces, with metrics *dX* and *dY*, is called *uniformly continuous* if for every ε > *0* there exists some δ *> 0* such that whenever *dX(x1,x2) <* δ for any *x1, x2* in *X*, we have *dY[f (x1), f (x2)] <* ε.

### Convexity

For the third mathematical property, it should be noted that convexity is defined in terms of *betweenness*:

*Definition 4*: A subset *C* of a space *S* is called *convex*, if whenever *x, y* are in *C*, then also all points between *x* and *y* are contained in *C*.

Betweenness can be defined for many non-metric spaces, for example for all graphs. An ordering structure also leads to a concept of betweenness, as will be discussed in a moment.

However, a metric space allows us to formulate notions of betweenness and thereby convexity in the following way:

*Definition 5:* Let *(S,d)* be a metric space. We say that *z* in *S* lies *between x, y* in *S* if *d(x,y) = d(x,z) + d(y,z).*

If the metric is Euclidean, this entails that *z* lies on the line between *x* and *y*.

Let us also observe that an order structure also leads to a concept of betweenness: Here, *z* is between *x* and *y*, if *x ≤ z ≤ y*. Note, however, that in contrast to the metric concept where *z* is between *x* and *y* if it is between *y* and *x*, betweenness derived from an order is asymmetric. If *x ≤ y* and *x ≠ y*, there might be points between *x* and *y*, but there cannot be any point between *y* and *x*.

When we have a map *f: X→Y* between metric spaces, we may require convexity preservation. This means that pre-images of convex sets be convex, or equivalently, that points *z* between *x* and *y* are mapped to points between *f(x)* and *f(y)*. Convexity preservation corresponds mathematically to the third constraint that intermediate forces lead to intermediate results. Depending on the geometry, this can be a very restrictive requirement. In Euclidean space of dimension *d ≥* 2 with its usual metric, this would imply that straight lines are mapped to straight lines, that is, the map is essentially linear. Thus, we need to restrict the underlying geometry for the concept to be useful.

We shall also speak of a *typical event* when the center of a region in force space is mapped to the center of a region in effect space. We recall here that we assume that these regions are convex, and therefore their centers tend to be sufficiently far away from their boundaries.

When we consider events as mappings *f: X→Y* between metric spaces, then the metric of the target space *Y* can be used to induce a metric on the event space, by *d(f,g):* = sup *d[f(x),g(x)]* where the supremum is taken over all *x* in (an appropriate region of) the domain *X*, and where the metric employed is that of *Y*. With such a notion of distance in an event space, we can then also introduce a notion of convexity in event space. An event *h* is then between events *f* and *g* if for all *x, h(x)* is between *f(x)* and *g(x)*. And as explained, if we have a notion of betweenness, we also obtain a concept of convexity. Note that this is different from the notion of convexity for a single event where we had required that the pre-image of a convex set be convex. Here, we speak about sets of events and define a notion of convexity in that context. That is, we no longer compare the application of different forces to the same events, but rather consider the effect of the same force in different events.

## Applications

The three mathematical structures that we have presented and their corresponding constraints on the mapping from force to result vectors generate a lot of inferences about events and event categories. Consequently, the structures that depend on events will be constrained by these inferences. We therefore believe that investigations of cognitive representations of events will be very fruitful in a range of domains.

### Event Structure and Semantics

Several of the semantic consequences of the event model are already presented in Warglien et al. (2012a) and Gärdenfors (2014). A model of an event can be a complex structure that not only involves the two vectors, but also a patient and an agent with their properties, as well as counter-forces, instruments, recipients, intentions, etc.

Even though the mental model of an event may be complex, a sentence normally captures only certain features of a construal generated from a particular focus on the event (see Croft and Wood, 2000, Ch. 3; Langacker, 2008, Ch. 3, for a survey). By analogy with the visual process – where we can only focus our attention on some features of the visual field – a construal focuses only on certain parts of an event. The sentences “Victoria hits Oscar” and “Oscar is hit by Victoria” describe the same event with the aid of two different construals, where Victoria and Oscar, respectively, are put in focus. Gärdenfors (2014, p. 177) proposes that a construal of an event contains as least one vector (force or result) and one object (patient or agent). Then he puts forward the thesis (ibid., p. 178) that a (declarative) sentence typically expresses a construal of an event.

This thesis about sentences implies that at least an agent or a patient (expressed by a noun phrase) and a force vector or a result vector (expressed by a verb phrase) are included in what is expressed. Thus the two main components noun phrase and verb phrase have to be present in a linguistic description of an event. The upshot is that given the event model and the thesis about construals, sentences are indeed central semantic units.

Some individual objects, in particular people and places, are given names in language. In contrast individual events are seldom represented in language. Exceptions are mainly historical events, e.g., the Flood, The French Revolution, the Second World War. However, event categories are often represented by nouns, e.g., birth, illness, marriage, football match.

After these general remarks about the role of events in semantics, let us now turn to the role of monotonicity, continuity and convexity in semantics.

The fundamental role of monotonicity from a semantic point of view is manifested in the ordinal nature of most modifiers of lexical categories related to events. This can be demonstrated by the use of adjectives and adverbs in modifying the assumed force and result vectors. In fact, many adjective pairs, e.g., “high”-“low,” “strong”-“weak,” “hot”-“cold,” “heavy”-“light,” only apply to ordered structures – so called *scalable* adjectives (see e.g., Paradis, 2001, 2008). Scalable adjectives also have *comparatives*: “higher,” “hotter,” “heavier” etc. The comparatives clearly depend on an ordering structure. Also for concepts with a prototypical structures, a partial ordering can be defined in terms of the distance to the prototype. Thus a color can be “greener” and a person can be “healthier.”

Adverbs typically exhibit ordinal properties as well. For example, the adverb “softly” in “Victoria hits Oscar softly” selects a sub-region of the force space where the value on the dimension of the strength of the force is relatively low (an ordering property). If monotonicity is assumed, then the value on the corresponding result dimension, i.e., pain, will also be low. So the inference in this case is that Oscar will only feel a mild pain (if any at all).

Next we come to the principle of continuity. Many verbs represent force or result vectors of events in which there is a continuous mapping between the two types of vector, for example, “heat,” “stretch” and “press.” As will be explained in Section “Manner and Result Verbs,” there are verbs that express violation of such continuity such as “cut” or “break” (Majid et al., 2007). Such verbs typically express the transition between only two possible states, that before the force is applied and that resulting from its application. A notable difference is that verbs expressing continuous events can be modified by adverbs that serve to express the degree of the effort or force applied. No such modification, however, is possible for verbs that express discontinuous phase transitions. For example one can “press strongly” (until something breaks), but one cannot “break strongly.” Of course, such transition verbs can be modified by adjectives addressing other dimensions. For instance, one can “break the window intentionally,” but already “breaking the window violently” requires some special context to be a viable expression.

Another difference is that verbs expressing continuous events the process are, in general, *reversible*, which is often represented by verb pairs such as push–pull, heat–cool, expand–shrink, etc. In contrast verbs for discontinuous transitions are not reversible: a stick that has been broken cannot be made “unbroken” and a child who is born cannot be “unborn.”

As regards the convexity constraint, Gärdenfors (2000, 2014) proposes that concepts are represented by convex regions in conceptual spaces. In particular actions can be represented as convex regions of patterns in force space (Gärdenfors, 2007; Gärdenfors and Warglien, 2012). Now suppose that you hear or read the sentence “Victoria hit Oscar.” Since you have no further information about the event, you interpret “hit” as a prototypical instance of hitting, i.e., close to the center of convex and continuous region of force space (or more generally action space) representing hitting. And from previous experience you know that this center is mapped into the interior of a region of pain space, for instance close to its center. In other words, you infer that hitting Oscar, or any other person for that matter, with a prototypical force, i.e., one located at the center of the force region, causes him pain. Since the pain region is also convex, its center prototypical area is located away from its boundary. Then, if the mapping from the force to the pain region is continuous, you assume that when Oscar is hit by Victoria the force is close to the prototypical one and you therefore conclude that the result will also be close to the center that part of the pain region.

The continuity and convexity constraints thus let us infer that when we are near a typical event then *similar actions produce similar results*, under the implicit ceteris paribus assumption.

A third example is Slobin et al. (2014), who studied how subjects named the actions shown in 34 video clips of different types of motion events such as walking, running and jumping, asking participants to name as specifically as possible the type of motion they just saw. Subjects were native speakers of English, Polish, Spanish, and Basque. A pairwise similarity matrix was created: for each participant and each pair of clips it was determined whether a pair of clips was called by the same term (1) or not (0). Then the individual participant matrices were summed to create a single language similarity matrix. Based on these matrices a two-dimensional multidimensional scaling solution was calculated. The solution shows for all languages four distinct, convex regions emerge corresponding to walking, running, crawling and to non-canonical motion (such as leaping or galloping). These results, and similar results from Malt et al. (2014), provide some support for the convexity thesis for event concepts.

## Manner and Result Verbs

In linguistics, it is common to distinguish between *manner verbs* and *result verbs* (see e.g., Rappaport Hovav and Levin, 2010; Levin and Rappaport Hovav, 2013). According to Rappaport Hovav and Levin (2010, p. 21) “manner verbs specify as part of their meaning a manner of carrying out an action, while result verbs specify the coming about of a result state.” Result verbs group together verbs describing motion with verbs that describe property changes because of the tendency to give the same linguistic construction to a changing entity as to a moving one: Both involve changes of properties, which the manner verbs do not. The distinction is supposed to be exhaustive (Levin and Rappaport Hovav, 2013): Any particular use of a verb is either a manner verb or a result verb (but not both).

On our event model the distinction comes out very naturally: manner verbs refer to force vectors of events while result verbs refer to result vectors. Another way of expressing this is to say that the manner/result distinction is basically a cause/effect distinction: manner verbs refer to causes and result verbs to effects.

When the monotonicity property can be assumed to hold, we can profile an event by either a manner or result verb without much loss of information. For example, when we say we pushed a table the default assumption is that some effect was produced, proportional to the intensity of the force exerted. Conversely, when using a result verb the default assumption will be that a force in the same direction was exerted by the agent (“I moved the table to the next room”). However, when such an assumption breaks down, we need to provide a more complete profile of the event, typically by introducing one more verb or suitable modifiers.

A follow-up question to the manner/result distinction is why there are no verbs that cover *both* the force and result vectors of an event. Our explanation, presented in Warglien et al. (2012a) builds on *learnability* constraints: Since the mapping between actions and results depend on counterforces and other contextual factors, such a mapping will be hard to learn and subject to many contingencies and sources of instability. For example, one’s understanding of the patterns of forces exerted by one’s arms is well integrated and the movement of an object in three dimensions is likewise integrated, but the relationship between the two is unstable, being subject to external counterforces and other uncontrollable factors.

A good example is when we have to account for the influence of counterforces. Mental models of events often contain counterforces such as supporting surfaces or friction, but their presence is in general not expressed. Important questions are then when and how counterforces can be expressed linguistically.

A general rule seems to be that counterforces are made explicit if and only if they prevent, obstruct or diminish the result expected from the application of the force under ordinary circumstances, or if they have the capacity to do so and therefore need to be overcome (Talmy, 1988). Linguistically, such breaks of expectation are typically expressed by ‘but’ constructions (Gärdenfors, 1994): “I pushed (manner) the coffin as hard as I could, *but* it did not move (result).” Sometimes, also facilitating forces can be expressed when they amplify the result beyond what is normally expected. In both cases, the additional forces move the result vector away from the prototype.

If you want to express a counterforce, it will function as a second cause and should be represented as a force vector. According to our account of events, you then need another event expression where the counterforce is the force vector. Consequently, linguistic expressions of counterforces will in general involve embedded clauses in a sentence that expresses the main event.

Talmy (1988) develops a schematic formalism that, for example, allows him to represent the difference in force patterns in expressions like “The ball kept rolling because of the wind blowing on it” and “The ball kept rolling despite the stiff grass.” In our terminology, the phrases “because of the wind blowing on it” and “despite the stiff grass” are embedded structures that express the secondary event based on the vector generated by a facilitating or a countering force. (To be precise, in the example above, the blowing wind is not a counterforce, but rather is a facilitating force since it goes in the same direction as the ball. Nevertheless it functions as a secondary force.)

Talmy’s (1988) analysis of force dynamics supports this conclusion. His dynamic ontology consists of two directed forces of unequal strength, the focal called “Agonist” (corresponding to what is called the force vector in the present article) and the opposing element called “Antagonist” (corresponding to the counterforce here).

A second way to express counterforces is to use modal verbs. Again, modal verbs involve another verb expressing a primary force (or the result of such a force). Many modal verbs relate to a counterforce. For example, in “I *let* the dog come in,” “let” expresses the removal of a counterforce (by opening a door). Or in “The dog may not come in,” the presence of a potential counterforce is made explicit.

A special case of causation occurs as a result of the *intentions* of an agent. The distinction between non-intentional and intentional sometimes shows up in result verbs. The classical case is *kill* versus *murder*. The latter is intentional, while the former is undetermined with respect to intentionality. Thus, *murder* cannot occur with non-intentional agents. Some events involving intentions, such as those describe by *give*, *buy* and *sell*, can be construed from either of two perspectives: physical causation or intentional causation leading to the fulfillment of a goal. Such a situation can still be expressed with the aid of a single verb, since the fulfillment of the intention presupposes a physical action, for example the transition of an object from an agent to a recipient.

### Contributions to Working Models and Segmentation

Psychological research on events has dedicated much attention to how complex flows of activities are segmented into simpler event entities by readers, observers or even direct participants (Zacks and Swallow, 2012). Our three constraints do not (and cannot) constitute a theory of such event segmentation, as we are here concerned with the structure of elementary, “atomic” events rather than with more complex event architectures. However, the three constraints may indirectly contribute to a better understanding of the segmentation process.

A central construct of psychological models of event segmentation is the working model. Event segmentation models submit that individuals hold at each moment (and for a given time scale) a single working memory representation of an event – what Zacks et al. (2007) define as the *working model* of the event. Working models of an event support the systematic effort of observers and participants to predict the future in response to current perception. Thus, working models need to be quite coarse and stable models of the event, a kind of static snapshots that are robust enough to remain buffered from the variations associated with the fine details of sensorial inputs (Radvansky and Zacks, 2014).

Event Segmentation Theory (Zacks et al., 2007) explains event segmentation as the result of updates in working models which respond to increases in predictive error of a current working model. According to the model, event boundaries correspond to such update activity. This entails that as long as the content of the working model does not violate the developments in the world, an event is continued.

We suggest that our three constraints may characterize the internal structure of working models and enrich the notion of what constitutes a “predictive error,” that is, a surprise. We thus consider our proposal as complementary to theories of event segmentation.

First of all, the three constraints clearly contribute to the buffering of working models from the details of perceptual inputs and to generate robust predictions. Monotonicity drastically simplifies causal inference, requiring only ordinal information on causes to infer ordinal properties of effects. Monotonicity helps to extrapolate and guide the prediction of causation in the working model. Continuity ensures that small changes in a neighborhood of effects are associated with small changes in causes. It thus predicts that observed variations in effects should be related to similar causes. Finally, convexity ensures that it is enough to know two event mappings to know what is in between them. Thus convexity helps to interpolate - for example to predict unspecified properties of the event. One could summarize by saying that the three properties ensure ordinal, topological and geometrical robustness to working models – and that they enable robust inference about causation, control of action and generalization. For example, in a narrative that only specifies a few snapshots of a story, the three constraints can help the reader or listener to fill in missing information.

Second, *violations* of three constraints provide a clear source of surprise that may lead to the update of a working model. Imagine that I am telling you that I made a trip from Venice to Paris. Then, for example, going to Lyon and then back to Milan before flying to Paris would violate monotonicity. Similarly, a long stopover at Milan would create a loss of continuity. Violations of convexity can also generate surprise. For example, if I am mentioning a trip from Venice to Paris, it would be surprising to find that the trip goes through Moscow.

Moreover, the three properties allow some natural forms of aggregation of elementary events. Monotonicity allows the reduction of multiple causal mappings into composite ones. For example the overarching event that somebody dies because he eats too much is a composition of the monotonicity of eating more leads to an increase in weight, which leads to increased coronary problems, which in turn leads to a higher probability of dying. Thus composition of events is facilitated by monotonicity. Convexity leads to a different types of aggregation, based on the creation of (convex) categories of events such as walking or running (Malt et al., 2014; Slobin et al., 2014) – as long as motion falls within a convex category, no changes in the motion component will be detected across elementary events. Continuity entails that even if a new event is initiated, many features of the previous event, such as the location of objects, do not change drastically (Magliano and Zacks, 2011).

## Discussion

In addition to our earlier work on event structure, we have in this paper introduced three general cognitive constraints on event mappings and shown how they can be translated into well-known general mathematical properties of mappings. The mathematical properties have broad implications for how we reason about events and for the semantics of natural language. Our approach unifies several areas since it brings out what is common to causal thinking, control of action and learning by generalization.

Human cognition does not seem to be plagued by the frame problem that has dogged the symbolic approach to AI, i.e., the problem how to describe the effects of actions succinctly without having to represent also all the trivial side effects or obvious non-effects of those actions. Rather, framing is a basic condition for perception and knowledge as such (Mammen, 2017). Apparently, humans use certain general principles to automatically infer (non-)consequences of actions, and those then no longer need to be represented explicitly. We believe that in this paper, we have identified three such principles, monotonicity, continuity and convexity.

While the three properties are presented in a specific model of events, they also apply to different accounts as long as they rely on some type of geometric representation. Our three constraints can be applied to the force dynamics of Talmy (1988), the causal models of Wolff (2007, 2008) and the event representations in Croft (2012). For example, the monotonicity constraint should apply whenever forces are analyzed in their causal role.

We conclude by a few notes on how the geometric approach relates to some of the problems concerning event representation that are discussed in the contemporary philosophical, linguistic and psychological debate.

Two main issues in such debates are lexical aspect (Aktionsart) and causality. We have not discussed aspect (Vendler, 1967), because it does not involve directly the mapping between the action and the effect spaces. However, we suggest that the typology of aspects follows more coherently from an ordinal treatment of time as a succession of (sub)events rather than by locating events on a Euclidean time line. Indeed, considering aspects in terms of bounds over the timeline (open/closed induces the telic non-telic distinction, while point vs. interval induces the instant/duration distinction) generates an anomaly: semelfactives, which are simultaneously non telic and instantaneous, would have to correspond to “open” (unbounded) points, which don’t exist in the standard topology of the line. On the other side, considering only ordered sequences of discrete time points allows to define duration as multiple points vs. instantaneous as single point, and telic vs. non telic as having or not having an end point. In this case a semelfactive just corresponds to a point which is not an end point (see Warglien et al., 2012a for a more detailed analysis of aspect).

Recently, there has been a strong growth of models of causal reasoning in terms of Bayesian networks. This approach has normative value, but it makes too strong claims about people’s ability to reason about probability distributions and to compute Bayesian updates. Sloman (2005, p. 103) claims that “people represent the qualitative structure of causal systems without accurately representing all quantitative details” (see also Sloman and Lagnado, 2015). In support of this, he notes that causal reasoning makes use of the monotonicity property: “On the flip side of the qualitative/quantitative divide, we do have access to some quantitative knowledge. We know that stepping harder on the accelerator pedal turns the wheels more than stepping lightly” (Sloman, 2005, p. 104). In addition, Waldmann and Hagmayer (2013) argue that people represent more aspects of the mechanisms of causality than the Bayesian formalism can encompass. The upshot is that we do not consider the Bayesian approach to be cognitively realistic. Our point is rather that the use of the general principles of monotonicity, continuity and convexity obviates the need for much of Bayesian reasoning.

On another front, our approach is resonant with the tradition starting from Davidson (1967) that associates events and causality. He writes that “events have a unique position in the framework of causal relations between events” (Davidson, 1967, p. 179). However, our model moves causality inside events. On our approach an event can become a cause of another event only as far its result vector can be transformed into a force vector. For example, when a driver turns the wheel in a car, her force results in a circular movement of the wheel. The driver immediately perceives that this result is transformed into forces that result in a change of the direction of the car. On our approach, this process involves two events, where the result vector of the first becomes the force vector of the second.

To turn to the favorite example of philosophers – billiard ball collisions – a good illustration of when the transformation from result vector to force vector breaks down is provided by Michotte’s (1963) experiments on the perception of collision events. By changing the angle of the trajectory of the second object after the collision, subjects clearly show a threshold beyond which the recognition of a causal event breaks down. This suggests that the subjects rely on an intuition of how trajectories translate into forces.

Our view of the role of causality in event structure is much closer to the one that has emerged in cognitive semantics. Like our approach, this tradition puts causality inside the event. It also emphasizes the paradigmatic function of two thematic roles, agent and patient, which are clearly connected to our two spaces (for a comparison see Warglien et al., 2012a). For example, Croft (2012) presents a geometrical model that shares many features with ours. However, a difference from our approach is that Croft does not develop the *force* aspect of the event (Warglien et al., 2012b). We believe that by taking into consideration the three constraints that have been analyzed in this paper, linguists will be able to explain several semantic aspects related to events, in particular in relation to the semantics of verbs. This may help to reconcile the two lives of events.

## Author Contributions

PG, JJ, and MW equally contributed to the conception and writing of the paper.

## Conflict of Interest Statement

The authors declare that the research was conducted in the absence of any commercial or financial relationships that could be construed as a potential conflict of interest.

## Footnotes

1 Although the notion of convexity can be generalized to non-metric spaces, as long as betweenness can be defined.

## References

[B1] Breidbach O. Jost J. (2006). On the gestalt concept. *Theory Biosci.* 125 19–36. 10.1016/j.thbio.2006.02.001 17046371

[B2] Caramazza A. McCloskey M. Green B. (1981). Naive beliefs in “sophisticated” subjects: misconceptions about trajectories of objects. *Cognition* 9 117–123. 10.1016/0010-0277(81)90007-X 7196821

[B3] Casati R. Varzi A. (2008). “Event concepts,” in *Understanding Events: From Perception to Action*, eds Shipley T. F. Zacks J. (New York, NY: Oxford University Press), 31–54. 10.1093/acprof:oso/9780195188370.003.0002

[B4] Croft W. (2012). *Verbs: Aspect and Causal Structure.* Oxford: Oxford University Press 10.1093/acprof:oso/9780199248582.001.0001

[B5] Croft W. A. Wood E. J. (2000). “Construal operations in linguistics and artificial intelligence, “in *Meaning and Cognition: A Multidisciplinary Approach*, ed. Albertazzi L. (Amsterdam: John Benjamins), 51–78.

[B6] Davidson D. (1967). “The logical form of action sentences, “in *The Logic of Decision and Action*, ed. Rescher N. (Pittsburgh, PA: University of Pittsburgh Press), 81–95.

[B7] Dijksterhuis E. (1950). *De Mechanisering van het Wereldbeeld.* Amsterdam: J.M.Meulenhoff.

[B8] diSessa A. A. (1983). “Phenomenology and the evolution of intuition,” in *Mental Models*, eds Gentner D. Stevens A. L. (Hillsdale, NJ: Lawrence Erlbaum Associates), 15–33.

[B9] Dowty D. (1991). Thematic proto-roles and argument selection. *Language* 67 547–619. 10.1353/lan.1991.0021

[B10] Duhem P. (1958). *Le Système du Monde, Tome VIII*, Paris: Hermann.

[B11] Ellis A. Young A. (1988). *Human Cognitive Neuropsychology.* Mahwah: L. Erlbaum.

[B12] Gärdenfors P. (1994). “The role of expectations in reasoning, “in *Knowledge Representation and Reasoning Under Uncertainty*, eds Masuch M. Polos L. (Berlin: Springer-Verlag), 1–16.

[B13] Gärdenfors P. (2000). *Conceptual Spaces: The Geometry of Thought.* Cambridge, MA: MIT Press.

[B14] Gärdenfors P. (2001). Concept learning: a geometrical model, *Proc. Aristotelian Soc.* 101 163–183. 10.1111/j.0066-7372.2003.00026.x

[B15] Gärdenfors P. (2007). “Representing actions and functional properties in conceptual spaces, “in *Body, Language and Mind* Vol. 1 eds Ziemke, T., Zlatev, J., and Frank, R. M (Berlin: Mouton de Gruyter), 167–195.

[B16] Gärdenfors P. (2014). *Geometry of Meaning: Semantics Based on Conceptual Spaces.* Cambridge, MA: MIT Press.

[B17] Gärdenfors P. Warglien M. (2012). Using conceptual spaces to model actions and events. *J. Semant.* 29 487–519. 10.1093/jos/ffs007

[B18] Gentner D. Kurtz K. J. (2005). “Relational categories, in *Categorization Inside and Outside the Laboratory*, eds Ahn W. K. Goldstone R. L. Love B. C. Markman A. B. Wolff P. W. (Washington, DC: American Psychological Association), 151–175.

[B19] Goldstone R. L. (1994). The role of similarity in categorization: providing a groundwork. *Cognition* 52 125–157. 10.1016/0010-0277(94)90065-5 7924201

[B20] Hume D. (1748/2000). *An Enquiry Concerning Human Understanding.* Oxford: Clarendon Press.

[B21] Jost J. (2015). *Mathematical Concepts.* Berlin: Springer Verlag 10.1007/978-3-319-20436-9

[B22] Kelley H. H. (1973). The processes of causal attribution. *Am. Psychol.* 28 107–128. 10.1037/h0034225

[B23] Langacker R. W. (2008). *Cognitive Grammar: A Basic Introduction.* Oxford: Oxford University Press 10.1093/acprof:oso/9780195331967.001.0001

[B24] Levin B. Hovav M. R. (2005). *Argument Realization.* Cambridge: Cambridge University Press.

[B25] Levin B. Rappaport Hovav M. (2013). “Lexicalized meaning and manner/result complementarity, “in *Subatomic Semantics of Event Predicates*, (Dordrecht: Springer), 49–70. 10.1007/978-94-007-5983-1_3

[B26] Magliano J. P. Zacks J. M. (2011). The impact of continuity editing in narrative film on event segmentation. *Cogn. Sci.* 35 1489–1517. 10.1111/j.1551-6709.2011.01202.x 21972849 PMC3208769

[B27] Majid A. Gullberg M. van Staden M. Bowermann M. (2007). How similar are semantic categories in closely related languages? A comparison of cutting and breaking in four Germanic languages. *Cogn. Linguist.* 18 179–194. 10.1515/COG.2007.007

[B28] Maier A. (1968). *Zwei Grundprobleme der Scholastischen Naturphilosophie.* Roma: Edizioni di Storia e Letteratura.

[B29] Malt B. Ameel E. Imai M. Gennari S. Saji N. M. Majid A. (2014). Human locomotion in languages: constraints on moving and meaning. *Mem. Lang.* 74 107–123. 10.1016/j.jml.2013.08.003

[B30] Mammen J. (2017). *A New Logical Foundation for Psychology.* Berlin: Springer 10.1007/978-3-319-67783-5

[B31] McCloskey M. Caramazza A. Green B. (1980). Curvilinear motion in the absence of external forces: naive beliefs about the motion of objects. *Science* 210 1139–1141. 10.1126/science.210.4474.1139 17831469

[B32] Michotte A. (1963). *The Perception of Causality.* London: Methuen.

[B33] Mill J. S. (1843). *A System of Logic.* London: John W. Parker.

[B34] Nosofsky R. M. (1988). Similarity, frequency, and category representations. *J. Exp. Psychol. Learn. Mem. Cogn.* 14 54–65. 10.1037/0278-7393.14.1.54

[B35] Paradis C. (2001). Adjectives and boundedness. *Cogn. Linguist.* 12 47–65. 10.1515/cogl.12.1.47

[B36] Paradis C. (2008). Configurations, construals and change: expressions of DEGREE. *English Lang. Linguist.* 12 317–343. 10.1017/S1360674308002645

[B37] Pianesi F. Varzi A. C. (2000). “Events and event talk,” in *Speaking of Events*, eds Higginbotham J. Pianesi F. Varzi A. C. (Oxford: Oxford University Press), 3–47.

[B38] Radvansky G. A. Zacks J. M. (2014). *Event Cognition.* Oxford: Oxford University Press 10.1093/acprof:oso/9780199898138.001.0001

[B39] Rappaport Hovav M. Levin B. (2010). “Reflections on manner/result complementarity, “in *Lexical Semantics, Syntax, and Event Structure*, Rappaport Hovav M. Doron D. Sichel I. (Oxford: Oxford University Press), 21–38. 10.1093/acprof:oso/9780199544325.003.0002

[B40] Richardson M. J. Marsh K. L. Isenhower R. W. Goodman J. R. Schmidt R. C. (2007). Rocking together: dynamics of intentional and unintentional interpersonal coordination. *Hum. Mov. Sci.* 26 867–891. 10.1016/j.humov.2007.07.002 17765345

[B41] Runesson S. Frykholm G. (1981). Visual perception of lifted weights. *J. Exp. Psychol. Hum. Percept. Perform.* 7 733–737 10.1037/0096-1523.7.4.733 6457088

[B42] Sewell W. H. (1996). Historical events as transformations of structures: inventing revolution at the Bastille. *Theory Soc.* 25 841–881. 10.1007/BF00159818

[B43] Slobin D. I. Ibarretxe-Antuñano I. Kopecka A. Majid A. (2014). Manners of human gait: a crosslinguistic event-naming study. *Cogn. Linguist.* 25 701–741. 10.1515/cog-2014-0061

[B44] Sloman S. A. (2005). *Causal Models: How we think about the World and its Alternatives.* New York, NY: Oxford University Press 10.1093/acprof:oso/9780195183115.001.0001

[B45] Sloman S. A. Lagnado D. (2015). Causality in thought. *Annu. Rev. Psychol.* 66 223–247. 10.1146/annurev-psych-010814-015135 25061673

[B46] Talmy L. (1988). Force dynamics in language and cognition. *Cogn. Sci.* 12 49–100. 10.1207/s15516709cog1201_2 28951449

[B47] Vendler Z. (1967). “Verbs and times, ” *Linguistics in Philosophy*, ed. Vendler Z. (Ithaca, NY: Cornell University Press), 97–121.

[B48] Waldmann M. R. Hagmayer Y. (2013). “Causal reasoning,” in *Oxford Library of Psychology. The Oxford Handbook of Cognitive Psychology*, ed. Reisberg D. (New York, NY: Oxford University Press), 733–752. 10.1093/oxfordhb/9780195376746.013.0046

[B49] Warglien M Gärdenfors P. Westera M. (2012a). Event structure, conceptual spaces and the semantics of verbs. *Theor. Linguist.* 38 159–193. 10.1515/tl-2012-0010

[B50] Warglien M Gärdenfors P. Westera M. (2012b). Replies to comments. *Theor. Linguist.* 38 249–264. 10.1515/tl-2012-0016

[B51] Wellman M. P. (1990). Fundamental concepts of qualitative probabilistic networks. *Artif. Intell.* 44 257–303. 10.1016/0004-3702(90)90026-V

[B52] White P. A. (2012). Visual impressions of causality: effects of manipulating the direction of the target object’s motion in a collision event. *Vis. Cogn.* 20 121–142. 10.1080/13506285.2011.653418

[B53] Wolff P. (2007). Representing causation. *J. Exp. Psychol. Gen.* 136 82–111. 10.1037/0096-3445.136.1.82 17324086

[B54] Wolff P. (2008). “Dynamics and the perception of causal events, “in *Understanding Events: How Humans See, Represent, and Act on Events*, eds Shipley T. Zacks J. (Oxford: Oxford University Press), 555–587. 10.1093/acprof:oso/9780195188370.003.0023

[B55] Wolff P. (2012). Representing verbs with force vectors. *Theor. Linguist.* 38 237–248. 10.1515/tl-2012-0015

[B56] Wolpert D. M. Flanagan J. R. (2001). Motor prediction. *Curr. Biol.* 11 R729–R732. 10.1016/S0960-9822(01)00432-8 11566114

[B57] Zacks J. Swallow K. M. (2012). Event segmentation. *Curr. Dir. Psychol. Sci.* 16 80–84. 10.1111/j.1467-8721.2007.00480.x 22468032 PMC3314399

[B58] Zacks J. Tversky B. (2001). Event structures in perception and conception. *Psychol. Bull.* 127 3–21. 10.1037/0033-2909.127.1.3 11271755

[B59] Zacks J. M. Speer N. K. Swallow K. M. Braver T. S. Reynolds J. R. (2007). Event perception: a mind-brain perspective. *Psychol. Bull.* 133 273–293. 10.1037/0033-2909.133.2.273 17338600 PMC2852534

